# Using the Primary care Academic CollaboraTive to explore the characteristics and healthcare use of older housebound patients in England: protocol for a retrospective observational study and clinician survey (the CHiP study)

**DOI:** 10.3399/BJGPO.2023.0114

**Published:** 2023-11-01

**Authors:** Elizabeth Winn, Madeleine Kissane, Samuel WD Merriel, Thomas Brain, Victoria A Silverwood, Ishbel Orla Whitehead, Laura D Howe, Rupert A Payne, Polly Duncan

**Affiliations:** 1 Centre for Academic Primary Care and Population Health Sciences, University of Bristol, Bristol, UK; 2 Centre for Primary Care and Health Services Research, University of Manchester, Manchester, UK; 3 School of Medicine, Keele University, Keele, UK; 4 Population Health Sciences Institute, Newcastle University, Newcastle upon Tyne, UK; 5 Exeter Collaboration for Academic Primary Care (APEx), Exeter Medical School, University of Exeter, Exeter, UK

**Keywords:** Primary Health Care, General Practice, House-bound Persons, Homebound Persons, Healthcare Utilisation, Collaborative Research

## Abstract

**Background:**

Older housebound people are an under-researched group for whom achieving good primary health care can be resource intensive.

**Aims:**

To describe the characteristics and healthcare use of older (≥65 years) housebound people; explore clinician views on delivery of care to housebound people; and assess the feasibility of using a new network of healthcare professionals to deliver high quality research.

**Design & setting:**

Retrospective observational study of electronic GP records and clinician survey in England.

**Method:**

Clinical members of a new UK research network called the Primary care Academic CollaboraTive (PACT) will collect the data. For part A, around 20 GP practices will be recruited and clinicians will identify 20 housebound and 20 non-housebound people, matched by age and gender (around 400 total in each group). Anonymised data will be collected on characteristics (age, gender, ethnicity, deprivation decile), long-term conditions, prescribed medicines, quality of healthcare (via Quality Outcomes Framework targets), and continuity of care. Reports with benchmarked practice-level data will be provided to practices to identify areas for quality improvement and to enhance engagement. For part B, 2–4 clinicians will be recruited from around 50 practices in England (around 150 clinicians) to complete a survey about delivery of healthcare for housebound people. For part C, data will be collected to assess the feasibility of using the PACT network to deliver primary care research.

**Conclusion:**

Older housebound people are a neglected group both in terms of research and clinical care. Understanding the characteristics and use of primary healthcare of housebound people will help identify how to improve their care.

## How this fits in

Little is known about the characteristics and use of primary healthcare by older housebound people. This study, using a novel collaborative research model, will collect data from practices across England on characteristics, long-term conditions, medicines, quality of care, and continuity of care of older housebound and non-housebound patients. Study findings will help identify priority areas for future research aimed at improving primary healthcare for older housebound people.

## Introduction

Housebound people (defined as those unable to attend the GP surgery, visited by a primary healthcare professional [HCP] at home) are an under-researched group.^1^ Research from the 1990s found that around 20% of people over 85 years were housebound^2,3^ (now around 340 000 people within this age group in the UK).^4^ Home visits are time-consuming and, due to high workload pressures, acute problems are possibly being prioritised over proactive care of long-term conditions, such as optimising medicines and advance care planning.

Unmet healthcare needs (the difference between healthcare that is deemed necessary and the actual care received)^5^ depend on the characteristics of patients seeking healthcare (for example sociodemographics) and the services available to them. A study in France found that unmet healthcare needs were strongly associated with being housebound.^6^ A qualitative study of marginalised UK communities found that housebound participants experienced significant problems accessing healthcare.^7^

Problematic polypharmacy, where the potential harm from medicines outweighs the potential benefit, is one example of unmet healthcare need.^8^ Medicine reviews offer an opportunity to improve prescribing safety by rationalising prescribing, simplifying regimens, and stopping or tapering down non-essential and potentially harmful medicines.^9^ GPs and pharmacists report difficulty in stopping medicines without involving the patient.^10^ GP home visits are generally focused on acute problems, and home-based pharmacist reviews are seldom provided, likely resulting in suboptimal medicine reviews for older housebound people.^11^

Better understanding the healthcare needs of the UK housebound population is essential for improving healthcare services. The undertaking of such research may benefit from novel strategies to improve reach and recruitment.

### Aims

The primary aim of the study is to describe the characteristics and primary healthcare use of older housebound people and to understand how they differ from non-housebound people.

The primary aim of this study is broken down into the following specific objectives:

1. To examine the characteristics and long-term conditions of older (≥65 years) housebound and non-housebound patients, including:
  - sociodemographic characteristics (for example age, gender, ethnicity, deprivation decile);
  - long-term conditions, including morbidity count and Cambridge Multimorbidity Score;^12^
  - frailty (Rockwood score).^13^
2. To compare healthcare utilisation of older (≥65 years) housebound and non-housebound patients, including:
  - prescribed medicines in the preceding 12-month period, including potentially problematic prescribing;
  - continuity of care;
  - quality of care;
  - number and type of in-hours and out-of-hours GP appointments;
  - and use of other healthcare services.
3. To explore clinician views on the delivery of care to housebound patients, including:
  - how care of housebound patients is organised and delivered;
  - and views on delivery of healthcare.

The secondary aim of the study is to assess the feasibility of using a new primary care network of trainees and HCPs (PACT) to carry out high quality research, including:

- to describe the number of PACT members who are interested and, of those selected, the proportion that complete data collection;
- to describe the characteristics of GP practices and PACT members;
- to test whether valid high quality data can be collected using the PACT model;
- to assess whether the PACT model introduces active participation in research to previously research naïve practices and staff.

## Method

### What is PACT?

PACT — the Primary care Academic CollaboraTive — is a new initiative that aims to build the capacity of UK academic primary care through engagement of trainees, GPs, and other HCPs (hereinafter PACT members) in high quality research focused on improving patient care.^14^ PACT members working in GP practices across the UK will collectively take part in projects. This innovative model of research will allow researchers to include patients who are difficult to identify by other research methods (for example via Clinical Practice Research Datalink), such as housebound patients, and to create bespoke datasets by combining data extracted from the GP records, patient surveys, and practice workforce surveys. As PACT members are clinically trained, they can carry out detailed case note reviews of the GP records, extracting information that is not captured in routinely collected coded data.

### Recruitment

The study will be advertised via the Clinical Research Network (CRN), PACT network (HCPs who have registered an interest in taking part in PACT projects), deaneries, social media, GP teaching newsletters, Royal College of General Practitioners Associates in Training network, and the research team’s local networks. The authors will purposively reach out to those working in more deprived and rural areas, as these patients are generally underrepresented in research.^15^ To register an interest, PACT members (who will collect data for the study) will complete an online expression of interest form, and a GP partner or practice manager will complete a practice agreement form.

For part A (observational study), around 20 GP practices using EMIS (electronic GP records) will be invited to take part from six CRNs (Box 1). For part B (clinician survey), around 50 GP practices from across England will be invited to take part. The authors will endeavour to select practices serving a range of sociodemographic populations (for example, urban and rural; deprived and affluent; different ethnic groups).

Box 1 **Eligibility criteria for GP practices for observational study (part A)**

- GP practice within the East Midlands, North East and North Cumbria, North West Coast, South West Peninsula, Yorkshire and Humber, and West of England CRNs
- GP practice uses EMIS software (electronic GP record)
- One or more foundation doctors, GP trainees, or clinicians interested and available to collect data for the study (four half days of work)
- Practice delivered home visits in April, May, and June 2022
- Written consent from a GP partner or practice manager giving permission for the practice to take part in the study and for the PACT member to use four half-days of protected study time for the project
- GP and/or nurse available to supervise the PACT member (2 hours total, cost reimbursed via the CRN service support costs)
- Practice administration team available to support the PACT member with administrative tasks (3 hours total, cost reimbursed via the CRN service support costs)

### Data collection

#### Part A (observational study)

Data collection will be piloted in a small number of practices prior to scaling up. Detailed standard operating procedures and ‘Frequently Asked Questions’ will be provided to PACT members. The study team will troubleshoot via email, phone, and videoconference calls. PACT members will complete online Good Clinical Practice training (3 hours of online research training).

PACT members will identify 20 housebound (who have had at least one home visit) and 20 non-housebound people (who have attended the GP surgery with no record of a home visit) aged ≥65 years with a face-to-face consultation in April to June 2022. Nursing home or residential home residents will be excluded. A pre-prepared electronic search will identify potentially eligible patients, using ‘home visit requested’, ‘home visit’ appointment slot, and ‘home visit’ codes for housebound patients and ‘GP surgery’ codes for non-housebound patients (Table 1).

**Table 1.** Patient eligibility criteria for observational study (part A)

	Inclusion criteria	Exclusion criteria
Cases	Visited at home by a clinician from the GP practice in April, May, or June 2022 ≥65 years on 1 April 2022	Living in a nursing home or residential home
Controls	Face-to-face GP surgery appointment in April, May, or June 2022 ≥65 years on 1 April 2022	Consultations for blood tests and observations (for example blood pressure checks) only Listed for a ‘home visit appointment’ and/or ‘home visit consultation’ recorded in notes in April, May, or June 2022 Living in a nursing home or residential home Coded as ‘housebound’ in the GP record

Each housebound ‘case’ will be matched by 5-year age group and gender to a non-housebound ‘control’ using a case–control matching tool, developed for the purpose of the study using Microsoft Excel software (version 365). The tool randomly sorts the cases and controls, and finds an age- and gender-matched control for each eligible case. The PACT member will review the electronic GP records to check eligibility, completing an online Case Report Form using REDCap software (version 3, Supplementary File 1).

Anonymised routinely collected data will be extracted using a pre-prepared search of the electronic GP records (Table 2). For all eligible cases and controls, the electronic GP record will be reviewed from 1 April 2021 to 31 March 2022, including uploaded documents. Anonymised healthcare use data, will be extracted using an online Case Report Form using REDCap (version 3) software (Supplementary File 2, Table 2). A study ID number will link manually extracted data to automated search data.

**Table 2.** Summary of anonymised patient variables to be collected for Part A (observational study)

Routinely collected data	
Sociodemographics	• Age • Gender • Index of multiple deprivation • Ethnicity
Long-term conditions	• Morbidity count • Presence of long-term conditions (from the validated list of 20 medical conditions included in the Cambridge multimorbidity score)^12^ • Cambridge multimorbidity score^12^ (abbreviated version including 20 long-term conditions, general outcome)
Measures of frailty and coding of housebound	• Rockwood frailty score^13^ • ‘Housebound’ problem code
Medicines	• Total number of medicines • Polypharmacy (≥5 or≥10 active repeat medicines) • Anticholinergic burden score^18^ • Medication regimen complexity index^19^ • Medication review of notes/with patient • High risk prescribing indicators (e.g. aspirin in a patient ≥65 years without a proton pump inhibitor)*
In-hour GP appointments	• Total number • Number of phone calls, GP surgery consultations, home visits, and video calls • Accuracy of coding of appointment type (e.g. phone call, home visit, etc)
Continuity of careQuality of care	• Using Bice-Boxerman index^20^ from coded in-hours consultations (all types) • Quality and Outcomes Framework (QOF) indicators
**Manually extracted data (from uploaded documents)**	
Out-of-hours primary care appointments	• Number of phone calls, on-site consultations, home visits, and video calls
Hospital utilisation	•Emergency department attendances (number) • Hospital admissions (type [elective/planned], length of stay) • Outpatient appointments (number)
111 calls and ambulance attendances	• Number of 111 calls and ambulance attendances
Community consultations (e.g. mental health, physiotherapy, occupational therapy, audiology, chiropody, optician, dental, etc)	• Number and type of consultation (e.g. mental health, physiotherapy, etc)

* The searches for high risk prescribing indicators were developed by Ardens Healthcare Informatics and are based on NHS Business Services Associate Medication Safety - Indicators Specification (August 2019) (Supplementary File 4).

#### Power calculation for part A

To estimate the difference between housebound and non-housebound people in morbidity count, the authors used the results of a study in the US (7603 adults ≥65 years):^16^ morbidity count 2.5 and 3.9 for non-housebound and housebound participants respectively (standard deviation 1.5). If the authors collect data on 200 housebound and 200 non-housebound people, this magnitude of difference could be detected with more than 90% power at the 5% significance level. If the authors collect data on 400 cases and 400 controls (the target), they will detect smaller differences with greater precision.

#### Part B (clinician survey)

PACT members will complete a short online survey about organisation of healthcare for housebound patients, including the workforce involved in home visits, the types of healthcare offered to housebound patients (urgent or non-urgent), and coding of housebound patients (Supplementary File 3). They will also recruit 2–4 clinicians (around 150 clinicians in total) to complete a 10-minute online survey (using REDCap software, version 3) about changes to the delivery of care to housebound patients since the COVID-19 pandemic, and challenges and innovative solutions to delivering care (Supplementary File 5).

#### Part C (feasibility study)

The authors will collect data about the characteristics of PACT members who express an interest in taking part and their practice populations (Supplementary File 6). Participating PACT members and their supervisors will complete short surveys to examine their reason and motivation for signing up, and their experience of taking part (Supplementary File 7). To test whether high quality data can be collected using the PACT model, data quality exercises will be completed in around five practices, whereby a second PACT member will independently check patient eligibility and manually extract healthcare use data.

### Analysis plan

#### Part A (observational study)

A descriptive analysis of the sociodemographic characteristics, long-term conditions, and prescribed medicines will be completed for housebound and non-housebound patients.

Regression analyses will be used to compare the characteristics and healthcare utilisation of housebound and non-housebound patients, by:

- examining the sociodemographic characteristics, long-term conditions, and frailty of older housebound and non-housebound people (outcome: housebound status); and
- comparing healthcare use of older housebound and non-housebound people using key outcome variables such as prescribed medicines in the preceding 12-month period, continuity of care, and number of in-hours GP appointments (outcome: healthcare use).

A summary of the analysis plan for Part A is shown in Box 2.

Box 2 **Summary of planned regression analyses for part A (observational study)**

Aim: to examine the characteristics and long-term conditions of older (≥65 years) housebound and non-housebound patients.

Groups to be compared:

- housebound or non-housebound.

Key characteristics to be compared:

- sociodemographic characteristics (age, gender, deprivation level);
- long-term conditions (morbidity count, Cambridge multimorbidity score,^12^ and prevalence of specific conditions);
- frailty (Rockwood score).^13^

Aim: To compare healthcare utilisation of older (≥65 years) housebound and non-housebound patients.

Key outcome variables:

- prescribed medications in the preceding 12-month period (for example total number of medications, rates of polypharmacy [≥5 or ≥10 medications], anticholinergic burden score,^18^ and medication regimen complexity index);^19^
- continuity of care;^20^
- number of in-hours GP appointments (phone calls, home visits, video consultations, GP surgery visits).

Exposure variables:

- housebound or non-housebound.

Covariates:

- age, gender, deprivation level,
- long-term conditions,
- frailty,
- GP practice.

#### Part B (clinician survey)

A descriptive analysis will examine the clinician survey data. Mean and standard deviation will be used to describe distribution for normally distributed data; median, interquartile range (IQR) for non-normally distributed data. A subgroup analysis will be conducted for the following groups: rural/urban practices, deprived/affluent, practice list size.

#### Part C (feasibility study)

Using a flow diagram, the authors will examine the number of GP practices that expressed an interest, were invited, and completed the project. A descriptive analysis will examine the characteristics of PACT members (for example age, gender, ethnicity, stage in training) and their GP practices (for example list size, deprivation decile, ethnicity estimates, urban versus rural); the reasons they chose to participate; and their experience of taking part (Supplementary File 6). A subgroup analysis will be carried out for the following groups: research-active or research-inactive practices, practice list size, urban or rural location, practice deprivation level. Error rates will be calculated using data from the data quality exercise.

## Discussion

### Summary

This study will use a novel primary care research collaborative to explore the characteristics and primary healthcare use of older housebound people and clinician views on delivery of health care.

### Strengths and limitations

This is one of two exemplar studies to test the feasibility of using a network of trainees and primary HCPs to conduct high quality research.^17^ Using clinical staff to extract otherwise difficult to reach data is a key proposed benefit of this model and evaluating how this works in practice is a vital part of the data collection. Due to inconsistencies in coding of housebound status, housebound patients are difficult to identify using routinely collected data. This study will address this issue as clinicians will review the electronic GP records to check eligibility. A possible limitation is that some PACT members will have little research experience. Detailed instructions and support will be provided. Furthermore, the data quality exercise will provide evidence about the reliability and reproducibility of the data. A final limitation is that, despite collecting a broad range of healthcare use data, the authors are unable to collect data about healthcare provided by the district nursing team.

### Implications for research and practice

At a local level, PACT members will be provided with practice-level data benchmarked against other practices and will be encouraged to discuss the data with the practice team to identify areas for quality improvement, such as interventions to optimise coding of housebound patients and to reduce high risk prescribing.

Nationally, this study will answer important questions about healthcare use for older housebound patients. Manually extracted data (for example from hospital letters) will be combined with coded data (long-term conditions and medicines), to create a rich and bespoke dataset. Data collected may inform changes to clinical practice and ideas for future research aimed at reducing unmet healthcare need in older housebound patients.
